# Supplementary material for: Are carbon emissions trading and green financial instruments synergistic? -Comprehensive quantitative research based on content analysis
Source: PLoS One. 2024 Mar 7;19(3):e0298601. doi: 10.1371/journal.pone.0298601 (PMC10919601; doi:10.1371/journal.pone.0298601)
Supplement: S1 Data — (ZIP) [file pone.0298601.s001.zip › Data/地级市Z_F工作报告.zip!/║■▒▒/╩«╤▀╩╨/└┤╫╘╣1⁄2╓┌║┼í╛┬φ┐╦╩2╛▌═°í┐_2009.docx]

各位代表：

　　现在，我代表市人民政府向大会作工作报告，请予审议，并请各位政协委员和其他列席会议的同志提出意见。

　　一、2008年工作回顾

　　过去的一年是极不平凡的一年。在市委的正确领导下，全市上下深入贯彻落实科学发展观，齐心协力，顽强拼搏，奋力抗击历史罕见的冰雪灾害，积极应对不断蔓延的金融危机，全力抢抓扩大内需的政策机遇，成功举办了第三届世界传统武术节，全面启动了 “三城联创”工作，全市经济在应对危机中保持了较快增长，社会事业在统筹协调中全面进步，城乡面貌大为改观，人民生活明显改善。预计全年实现生产总值480亿元，同比增长10%；地方财政一般预算收入19.89亿元，增长27.6%；全社会固定资产投资180亿元，增长27%；社会消费品零售总额210亿元，增长23%；利用外资4800万美元，增长16.7%；外贸出口1.8亿美元，增长118.7%；城镇居民人均可支配收入10370元，增长10%；农民人均纯收入2841元，增长14.1%；万元生产总值综合能耗下降4.4%；城镇登记失业率为4.3%；人口自然增长率控制在6‰以内；居民消费价格总水平上涨7%。除物价外，市三届人大四次会议确定的主要目标全面完成。

　　（一）经济实现了持续较快增长。

　　农村经济稳步发展。实现农业增加值50亿元，增长5%。粮油产量分别增长8.3%和26.8%，连续五年丰收。新建特色基地20万亩，新增省级重点龙头企业1家、市级重点龙头企业20家，新增万头猪场11个、畜禽养殖小区200多个，茶叶和柑桔产量分别增长40%以上，水产品增长13.6%。实现劳务收入36.5亿元，增长14.6%。减少“两类人口”8万人，搬迁扶贫1.2万人。新修通村水泥路1640公里，治理水土流失265平方公里，完成4座大中型水库除险加固主体工程，争取低丘岗地改造项目9个、资金2亿元，解决了15万人饮水安全问题，新建沼气2万户、节柴灶4万座。

　　工业经济难中求进。全力支持东风公司提标稳产，着力实施双亿工程和中小企业成长工程，积极应对汽车排放标准提高和金融危机的双重影响，成立中小企业家联谊会、银企发展促进会和政信投资担保公司，千方百计帮助企业解决生产经营中的困难，遏制工业增幅回落势头。工业总量进一步扩大，规模以上工业增加值突破200亿元，增长10%，规模以上工业企业增加169家。结构进一步优化，地方工业占比48%，提高了7个百分点；50家“双亿”企业实现产值160亿元，增长33%；县域工业总产值增长27%，比全市平均水平高13个百分点。质量进一步提高，规模以上工业实现利润45.5亿元，增长19.2%；我市汽车产业集群荣获“中国产业集群品牌50强”称号。新的增长点进一步显现，ZD30轻型发动机投产，东风渝安、双星东风、东风特汽客车等扩能项目竣工，5000亩建设用地整理项目基本完成场地平整。

　　第三产业持续繁荣。实现增加值200亿元，增长14%。农村消费品零售总额增长25%，超过城市2个百分点。汽配城年交易额突破百亿大关；火车站商贸经济圈完成销售额52.3亿元，增长45.2%；云燕农博园一期工程开工；新建农村超市、农资放心店各100个。城区至武当山景区高速客运专线开通，武当山南神道景区对外开放，玉虚宫一期工程完工，太极湖新区开工建设。全市接待国内外游客921万人次，实现旅游收入41.8亿元，分别增长 21.2%和32.2%，其中武当山分别增长20.6%、88%。

　　财政金融稳健运行。财政收入增长超出预期，全年实现地方财政总收入44.86亿元，增长19.5%；地方财政一般预算支出71.41亿元，增长31.6%。金融机构各项存款余额573亿元，比年初增加98亿元；各项贷款余额235亿元，比年初增加48亿元。

　　（二）重点项目建设成效显著。

　　交通建设取得重大突破。武当山机场前期工作已经启动，襄渝铁路复线建设顺利推进，十宜铁路完成预可研报告。十房高速公路可行性报告通过评审，郧十、十白、谷竹高速公路完成可研报告。武神一级路改扩建工程十堰段全线开工。柳树垭隧道投入运营，土地岭、五条岭、雷锋垭隧道即将竣工。郧县汉江二桥、丹江习均大桥动工建设。汉江航道整治进展顺利。

　　水利电力建设全面推进。丹江大坝加高工程完成过半，潘口电站即将实现大坝截流，三里坪、周家垸电站建设进展顺利，龙背湾、小漩、孤山电站前期工作全面展开。120万千瓦热电联产项目纳入省“十一五”规划，500千伏输变电工程投入运行。

　　“三城联创”全面启动。丹江路、许白路建成通车，上海路一期工程动工，城区24条道路得到改造升级。市行政中心和接待中心投入使用，高速公路客运站开工建设。市容环境卫生专项整治力度加大，城市面貌焕然一新。我市通过全国创建文明城市工作先进城市验收，蝉联省级文明城市“五连冠”。国家环保模范城市26项考核指标已达标19项。城区、竹山县城、竹溪县城、浪河镇、宝丰镇、上津镇获得“楚天杯”奖，竹山县、市太和医院、习家店镇分别获得全国文明县城、全国文明单位和全国文明村镇荣誉称号。

　　项目争取工作初见成效。抢抓国家扩大内需政策机遇，成功申报和争取了一大批项目。共向国家发改委上报项目50多项，总投资326亿元；《丹江口库区及上游地区经济社会发展规划》和机场、铁路、公路、天然气等一批重大项目得到国家和省大力支持。

　　（三）改革开放深入推进。

　　各项改革继续深化。农村综合配套改革顺利通过省考评验收；集体林权制度改革全面铺开，确权发证占任务的67.9%；农村金融改革扎实推进，9家农村信用联社兑付中央银行专项票据5.2亿元，进一步明晰了产权关系，完善了法人治理结构。国有资产监管体制改革取得突破，市政府授权履行出资人职责的国有企业逐步到位，企业国有资产监管步入规范化轨道。企业改制工作顺利推进，市华中设计院、建筑设计研究院重组到位，新组建的城市公交公司运营良好。财政四项改革继续深化，公共财政体系日益完善。人事制度改革进程加快，事业单位合同管理用人机制初步建立。

　　对外开放取得新突破。实际利用外资大幅增长，增幅达16.7%；利用市外境内资金35亿元，增长10%。外贸出口增长118.7%，地方企业出口首次突破1亿美元大关。外派劳务增长12%，竹山县被命名为“全国外派劳务基地县”。

　　“两节”成功举办。69个国家和地区、2000余名运动员、3700多名嘉宾参加了第三届世界传统武术节（第八届武当国际旅游节），是我市有史以来举办的规模最大、规格最高的国际盛会。期间，投资贸易活动取得丰硕成果，签订合同项目28个，总投资40亿元。人民日报、中央电视台、凤凰卫视等37家境内外主流媒体，集中报道了我市经济社会发展情况和武术节盛况，极大地提升了十堰和武当山的影响力与美誉度。

　　（四）社会事业全面进步。

　　科教事业长足发展。新增高新技术企业8家，共申报专利560项，获省科技进步奖7项，省重大科技专项“黄姜皂素联产加工项目”开工建设，我市被国家科技部、公安部授予“全国科技强警示范城市”。素质教育全面推进，小学和初中入学率达100%；高中教育阶段普及率为82%，高于全省平均水平4个百分点；高考本科上线人数首次突破万人大关，增长51.5%；中职生就业率达98%。6.9万名城乡贫困生得到资助，7万名城市义务教育阶段学生首次免除学杂费。200所农村中小学改水改厕工程顺利完工，十堰职业技术学院成功搬迁，郧阳师专迁建一期单体工程完工，湖北汽车工业学院、郧阳医学院扩建工程进展顺利。

　　文体事业蓬勃发展。武当武术进机关、进学校、进社区等群众性文体活动深入开展，道教乐团在我市成立，现代豫剧《乡试》在首届湖北地方戏曲艺术节获奖，武当庙会等3个非物质文化遗产列入国家级保护项目。建成标准“农家书屋”232家，改造乡镇综合文化站8个，为100个行政村配备了体育设施。完成了1107个自然村广播电视工程建设，全市广播电视覆盖率进一步提高。

　　医疗卫生服务体系更加健全。新型农村合作医疗参合率达91.7%，比上年提高6个百分点。完成17个乡镇卫生院和200个村卫生室建设任务。有效应对了手足口病和奶粉事件，卫生应急工作在全省年度考核中名列第一。低生育水平保持稳定，人口出生率下降0.33个千分点，符合政策生育率提高0.86个百分点。

　　就业和社会保障体系进一步完善。城镇新增就业4.1万人，下岗失业人员再就业1.5万人。社会保险扩面新增9.86万人。城市低保补助标准由80元提高到128元，城乡居民享受低保人数分别达9.1万人和12.4万人。住房公积金制度基本建立，缴存职工达到24万人；发放廉租房住房租赁补贴7000多户，对人均住房面积10平方米以下的低保家庭实现了应保尽保，开工建设经济适用房15万平方米。完成雪灾倒房恢复重建和农村危房改造6000多户。13个国办福利院建设陆续开工。

　　生态建设和节能减排扎实推进。造林绿化30.3万亩，森林覆盖率达52.7%。神定河污水处理厂改造工程开工建设，丹江口库区生态环保项目全面推进。5家落后产能水泥厂、2家小钢铁企业、14家小电镀企业被关停，30家重点排污单位纳入实时监控，主要污染物排放总量继续下降，超额完成省定目标，我市荣膺省污染减排优秀单位。

　　社会大局和谐稳定。加大社会治安综合治理力度，依法打击和有效防范各类违法犯罪活动，治安防范机制逐步完善，全市安装视频监控探头3万多个，社会治安秩序良好，市公安局被中央政法委评为“集中排查化解涉法涉诉信访问题先进集体”。强化安全生产监管，受伤人数、死亡人数和直接经济损失分别下降18%、12.8%和5.1%。健全信访工作机制，注重从源头上化解矛盾，信访总量、进京非正常上访、赴省上访分别下降3.8%、49.5%、7.6%。

　　（五）民主法制建设进一步加强。

　　坚持定期向人大报告工作、向政协通报情况，重大决策征求民主党派、党外人士、人民团体意见，办理人大代表建议176件，办理政协委员提案332件，办复率100%。扎实开展了文明执法教育活动，行政复议案件结案率90%，行政审批事项办结率94.9%。坚持政务公开，123家公用事业单位推行了办事公开制度，1778个行政村村务全部公开，529家重点企业实行了厂务公开，市长热线受理群众反映问题的反馈率达100%。加强基层民主建设，第七届村(居)民委员会换届工作圆满完成。继续纠正行业不正之风，对市直10个单位进行了民主评议。组织社会捐款4000多万元支援四川地震灾区，完成了援川援疆工作年度计划。积极开展国防教育和“双拥”活动，民兵预备役和国防动员工作取得新进步，军政、军民关系进一步密切。着力解决人民群众关心的热点难点问题，2008年确定的十件实事全部完成。

　　在全市经济社会建设中，统计、审计、物价、供销、外事侨务、民族宗教、档案、地方志、邮政、地震、气象、水文、人防、对台、老龄、残联等工作都取得新的成绩。

　　各位代表，上述成绩的取得，是市委正确领导的结果，是市人大、市政协全力支持的结果，是全市人民共同奋斗的结果。我代表市政府，向所有参与、支持、关心十堰发展的同志们、朋友们表示衷心的感谢和崇高的敬意！

　　在总结成绩的同时，我们也清醒地认识到，我市经济社会发展中还存在不少问题：一是受汽车排放标准提高和金融危机的双重影响，以汽车工业为主导的全市工业增幅明显回落，企业订单减少、库存增加、资金链条吃紧。二是农业基础脆弱，产业化水平不高，农业增效、农民增收仍然比较困难。三是就业再就业压力较大，部分群众生活困难，社会保障任务依然很重。四是少数部门和部分公务人员在服务意识、执行能力以及廉洁自律等方面还存在一定的问题，政府自身建设需进一步加强。对于这些问题，我们将予以高度重视，积极采取有效措施，认真加以解决。[FS:PAGE]

　　二、2009年工作思路和主要措施

　　今年是实施“十一五”规划的攻坚年，做好今年的工作，具有十分重要的意义。我们应清醒地看到，新的一年是我市发展压力空前、困难空前、挑战空前的一年：国际金融危机继续扩散和蔓延，不仅本身尚未见底，而且对实体经济影响正在加深，其严重后果还会进一步显现，将使我市经济运行压力增大；国Ⅲ排放标准的实施，对汽车工业技术改造升级、节能减排达标和零部件企业配套能力提升提出了更高的要求，将使我市汽车工业发展困难增多；3.6万人的移民搬迁，7万人的脱贫，大量返乡农民工的安置就业，将使我市改善民生、维护稳定的任务更加艰巨。在看到困难和挑战的同时，也要看到我市仍然处在加快发展的重要战略机遇期，面临的重大机遇和有利条件前所未有：国家实施积极的财政政策和适度宽松的货币政策，一系列扩大内需、刺激经济增长的措施陆续出台，有利于我们争取项目和资金，有利于商用车市场回暖；党的十七届三中全会提出了新一轮农村改革发展的总体思路，加大了强农惠农力度，有利于我们破解“三农”难题、加快城乡一体化进程；《丹江口库区及上游地区经济社会发展规划》即将出台、《丹江口库区及上游水污染防治和水土保持规划》正在修编、丹江口库区生态补偿工作已经启动，有利于我们争取更多优惠政策，加快水源区发展；境外和沿海产业加快转型升级、加速向内地转移，有利于我们招商引资，承接产业转移；鄂西生态文化旅游圈的构建，有利于我市突破性发展生态文化旅游业，打造旅游经济强市；机场、铁路、高速公路、电站等一批重大项目的启动和建设，将使我市进入新一轮投资高峰期，经济增长的动力更加强劲；第三届世界传统武术节的成功举办，极大地鼓舞了人心，提升了城市形象，有利于我们进一步凝聚方方面面的智慧和力量，加快发展步伐。我们要进一步认清形势，把握大局，敢于迎难而上，坚定信心保增长；进一步解放思想，更新理念，善于抢抓机遇谋发展；进一步深化改革，扩大开放，创新机制，增强发展活力；进一步抓好项目建设，打基础管长远，夯实发展后劲；进一步加快结构调整，转变发展方式，提升经济内在素质；进一步统筹城乡发展，推进城乡一体化进程；进一步改善民生，提高人民群众生活水平和质量，促进和谐稳定，努力实现经济社会又好又快发展。

　　2009年经济社会发展的总体要求是：全面贯彻党的十七大和十七届三中全会精神，以邓小平理论和“三个代表”重要思想为指导，深入贯彻落实科学发展观，着眼于保增长促转型、保稳定促和谐，突破性发展农业产业化，突破性发展地方工业，突破性发展生态文化旅游业，加快构筑立体交通网络，全力打造区域性中心城市。

　　2009年全市经济社会发展主要预期目标是：生产总值增长10%；全社会固定资产投资增长30%；社会消费品零售总额增长20%；地方一般预算收入增长15%；利用外资5000万美元；外贸出口总额1亿美元；城镇居民人均可支配收入增长8%；农民人均纯收入增长8%；城镇新增就业3.5万人，登记失业率控制在4.5%以内；居民消费价格总水平、人口自然增长率、万元生产总值综合能耗、主要污染物排放量控制在省定目标以内。

　　围绕上述目标，我们将着力抓好七个方面的工作：

　　（一）以农业产业化为龙头，扎实推进新农村建设。

　　认真落实党的十七届三中全会精神，加大强农惠农力度，突破性发展农业产业化，千方百计增加农民收入，力争农业增加值增长6%，农民人均纯收入增加200元以上。

　　主攻产业化，打好优势牌。坚持以调整结构为主线，以培育龙头企业为突破口，以完善服务体系为支撑，实现产业与市场对接、工业与农业对接、农户与基地对接、科技与项目对接，不断提升产业化水平。一是围绕品牌抓基地，做大优势产业。进一步整合资源，加大无公害、绿色、有机食品品牌的培植和认证力度，着力打造金神龙烟叶、武当柑桔、神农本草中药材、房陵燕耳、马头山羊、丹江鲌鱼等十大品牌。围绕品牌，改造特色产业基地25万亩，重点调优10万亩柑橘和5万亩茶园；高标准新建烟叶、中药材、高山反季节蔬菜等特色产业基地20万亩；确保新增山羊、生猪、肉鸡等畜牧养殖小区100个，实现品牌优、产业强、规模效益好。二是围绕增值抓加工。支持现有龙头企业面向市场、依托资源做大做强，着力发展以雨润为龙头的肉鸡系列、以龙王垭为龙头的茶叶系列、以聚达食品为龙头的食用菌系列等精深加工业。千方百计培育和引进一批龙头企业，大力推进干鲜果、中药材、特色粮油产品及酒类等加工业发展。力创国家级重点龙头企业1家、省级重点龙头企业2家以上。三是围绕产销抓服务。健全公益性科技服务体系，加快优良品种的培育和引进，加大科技成果推广和转化力度。健全中介服务体系，力争新增农民专业合作组织30个。健全信息服务体系，及时发布气象、农业科技、农产品价格信息，提高农业产业化信息服务水平。

　　进一步抓好基础设施建设和惠农政策落实两大重点。一是加强农村基础设施建设。治理水土流失面积300平方公里，争取新开工4座中型水库、24座重点小型水库除险加固工程，切实抓好8个“民办公助”小农水项目和5个万亩灌区配套工程建设。启动7个低丘岗地改造项目，推进库区移土培肥工程建设。新建农村户用沼气4万户，改建节柴灶3万座。二是加强各项强农惠农政策的落实。深入贯彻《中共中央关于推进农村改革发展若干重大问题的决定》，及时兑付粮食直补、水稻良种补贴、农机购机补贴等各项强农惠农资金，认真落实国家扶持油料、稳定生猪生产等政策，着力减轻农民负担，最大限度地保护好、调动好农民的积极性。

　　大力破解农村发展和稳定的三大难题。一是进一步探索整村推进模式，破解扶贫开发难题。坚持开发式扶贫方针，以增加收入为中心，以整村推进为重点，力争减少贫困人口7万人，确保2007年111个重点村通过省市验收，完成2008年实施的109个重点村建设任务，启动170个重点村整村推进工作。全面完成城区农村扶贫开发五年规划，实现“村通水泥路、组通砂石路、户饮洁净水、通信电视全覆盖”目标。二是精心组织，破解移民安置难题。认真落实移民政策，及时解决移民生产生活中的困难和问题，力争完成南水北调1万移民外迁试点、潘口电站2.4万移民搬迁安置任务，确保移民迁得出、稳得住、能致富。三是拓宽渠道，破解返乡农民工就业难题。加快建立城乡统一的人力资源市场，组织返乡农民工参加阳光工程培训、回归创业培训，积极引导农民工实现再就业，通过特色产业基地建设吸纳一批，农村基础设施建设消化一批，农副产品加工业转化一批，发展特色劳务转移一批，力争新转移农村劳动力3万人。

　　（二）以工业为主导，打造工业经济强市。

　　坚持以汽车工业为龙头，以双亿工程为抓手，以工业园区为载体，不断优化工业经济结构，推进企业自主创新，千方百计服务企业，进一步夯实工业主导地位，力争实现工业增加值220亿元，增长10%；地方工业增幅高于全市平均水平。

　　进一步做优汽车产业集群。重点抓整车，提升零部件，构建大基地，千方百计做优产业集群。一是以东风公司为龙头，实施品牌战略，大力发展重型车、轻（微）型车、客车、改装车四大系列整车产品。支持东风商用车在稳产保销的同时，加快提升客车产能和重型车综合生产能力。重点支持东风渝安15万辆微型车、东风特汽5000辆客车、三环专汽3万辆整车、世纪中远5000辆轻型车、驰田露天矿山重型自卸车等扩能改造项目建设，力争全年整车产销量达到30万辆以上。二是以关键零部件为重点，大力发展发动机、变速箱、车身、车架、车桥等五大总成。重点支持东风ZD30发动机、正和4万台车身、运意5万套车桥等扩大产能，支持东风11升发动机、4H发动机项目尽早开工。鼓励汽配企业围绕总成积极开发配套产品，重点支持先锋大型模具及数控机床开发、双星东风1000万套轮胎、普林30万套主被动齿轮、同创工贸120万套同步齿环、天涯科技100套汽车空调、三立灯具系列车灯、郧县佳恒系列液压产品、天运汽车电子等项目建设，以总成为纽带形成序列化、标准化、集约化的汽配产业群。三是以汽配专业市场为载体，打造国家级汽配产业基地和汽配交易中心。大力支持中国（十堰）汽配城进一步完善功能、扩大规模，确保销售额增长10%以上。

　　全力实施“四大工程”，鼓励企业成长壮大。一是实施全民创业工程。健全社会化服务体系，降低创业门槛，激发全民创业热情，确保新增各类中小企业1000家以上。二是实施中小企业成长工程。选择100家成长性好、竞争力强的中小企业，优先扶持和重点培育成为规模以上企业。三是继续实施双亿工程。鼓励规模以上企业加大研发投入，加大技改投入，加大市场投入，发展成为新的双亿企业，力争产值过亿元的企业新增20家、过5亿元的企业新增5家、过10亿元的企业新增3家。四是实施企业提升工程。积极指导汽车及零部件生产企业科学有序发展，提升整车生产水平，整合关键零部件生产，重点扶持100家汽车零部件生产企业增强核心竞争力，成为行业骨干和中坚，优化企业结构。

　　大力发展县域工业。县域经济必须坚持以工业为主导。一是突出做好市场前景好、资源潜力大的农副产品深加工这篇大文章，打造特色鲜明的绿色食品加工业。二是十分注重合理开发利用中药材资源，促进生物医药产业的调整与发展。三是大力推进水电开发，加快潘口电站建设步伐，开工建设龙背湾、小漩、夹河关、白沙水电站，争取孤山电站立项。四是科学有序推进矿产资源开发，提高矿产品深加工水平。要高度重视抓工业，千方百计抓工业，确保县域工业增幅高于全市平均水平10个百分点以上。

　　加快开发区和工业园区建设步伐。做好十堰经济开发区跨域发展战略规划的基础性工作，努力拓展发展空间。做好万亩山地整理项目的区域功能划分和产业布局规划，尽快启动“四通”（即通路、通水、通电、通信）配套基础设施建设，为尽早吸纳项目入驻创造条件，发挥效益。加快温州汽配、普林二期、东风孵化、东风汽车电子等园区建设步伐，吸纳优势企业入驻，使园区真正成为招商引资的主阵地、产业集聚的新高地、工业发展的增长极。

　　全面提升市场主体素质。强化“工业第一、企业家老大”的理念，大力实施企业家素质提升工程。积极搭建企业家协会、研修班等互动交流学习平台，激励经营管理者提高自身素质和驾驭市场的能力，尽快实现从普通经营者到企业家的跨越。引导企业创建学习型企业，加强教育培训，提高员工素质。鼓励企业更新发展理念，增强策划意识，塑造企业文化，推进自主创新，苦练内功，为下一轮发展积蓄力量。

　　千方百计帮助企业解决现实困难。把保企业生存作为首要任务来抓，动员一切力量，集聚一切资源，帮助企业增信心、减负担、度难关。一是着力解决中小企业融资困难。鼓励金融机构抓住国家实行适度宽松货币政策的机遇，加大金融产品创新力度，推进小额贷款公司和村镇银行试点工作，扩大信贷投放规模。进一步强化财政性资金的引导作用，多方筹集资金，全力支持企业发展。充分发挥中小企业家联谊会、银企发展促进会和政信投资担保公司的作用，积极探索信用担保、中小企业联保、产业链联保等新的担保方式，力争各类担保资本金达到2亿元、担保金额达10亿元以上。加强对评估机构的监督管理，规范评估操作行为，降低企业融资成本。帮助企业解决上市前期费用，支持市属企业上市融资。二是着力解决企业急需用地问题。加强土地调度，优先保证市场前景好、成长性强、投资强度大、技术含量高的企业用地需要。支持企业入驻工业园区，集约节约用地，提高土地使用效率。实行一企一策、特事特办，尽快完善企业土地和房产手续。三是着力减轻企业负担。全面落实《支持城区加快新型工业化发展若干政策规定》，坚决兑现优惠政策。调整土地等级范围，降低企业用地成本。加大清费治乱减负力度，进一步调动企业发展的积极性。

　　（三）突破性发展生态文化旅游业，促进三产提档升级。

　　高标准、大手笔打造生态文化旅游核心板块。充分发挥大山、大水、大人文的优势，以武当山为龙头，以“三区”（武当山核心区、丹江口水源区、十堰城区）、“三线”（武当山至丹江东部线、武当山至两郧西部线、武当山至房县南部线）为开发重点，努力把十堰建成鄂西生态文化旅游圈的核心板块和重要支撑。确保2009年全市接待游客人数和旅游总收入均增长15%以上。一是在规划编制上实现新突破。瞄准国际先进水平，立足全国、全省旅游发展格局，高起点、高标准修订《十堰市旅游发展总体规划》，抓好丹江口库区生态旅游规划和武当山新区发展规划的编制，加快建立由市到县的多层次旅游发展规划体系。二是在基础设施建设上实现新突破。加快推进市游客服务中心、玉虚宫维修、太极湖新区、白马山旅游区配套设施、国家地质公园、龙潭河、庐陵王故居开发等旅游项目建设，完善景区景点的配套设施。三是围绕“三区”“三线”，在精品线路打造上实现新突破。注重发挥旅行社和导游的宣传促销主导作用，积极开发旅游主打产品，深度挖掘和包装武当文化，增强游客参与性和互动性，把“宗教朝圣游、武术修学游、仙山秀水游、城郊生态休闲游、古文化探秘游”等线路打造成为旅游热门线路。四是在旅游商品开发上实现新突破。鼓励企业和民间手工艺者针对不同层次游客需求，大力开发绿松石、银制品、武当武术及宗教用品、地方土特产品系列，深度挖掘文化内涵，彰显地域文化特色，提高市场竞争力。五是在体制机制创新上实现新突破。创新投入机制，建立旅游发展基金，从今年起市财政每年拿出专项资金2000万元，支持旅游产业发展；大力支持武当山组建集团，争取上市融资。创新景区经营管理体制，实行旅游资源资本化、景区经营公司化，创造条件成立旅游发展总公司或旅游管理服务中心，促进旅游集约化经营，确保景区开发规范有序、资源利用科学合理。

　　提升商贸流通业发展水平。围绕吃、住、行、游、购、娱六大旅游要素，优化商业网点布局，加快认定旅游定点饭店、定点购物商店、定点娱乐场所，打造特色餐饮区、综合购物区、休闲娱乐区。围绕服务现代工业，加快打造火车站商贸经济圈、汽车汽配物流园、汉江物流园三个百亿物流园。围绕培育商贸领军企业，重点支持人民商场、寿康永乐、京华超市等一批大企业，发展连锁经营，扩大市场份额。围绕扮靓城市景观，重点改造提升五堰步行街、邮电街等临街业态。围绕扩大农村消费，深入推进“万村千乡”市场工程、“家电下乡工程”，新建和改造一批农家店和农村商品配送中心，健全农村市场流通体系，挖掘农村消费潜力。

　　大力发展新兴服务业。重点发展技术开发、营销策划、售后服务、人才培训、电子商务、信息咨询、创意设计和服务外包等生产性服务业，促进制造业提升核心竞争力。加快发展社区服务、休闲娱乐、体育健身、医疗保健等面向民生的服务业，促进消费升级。[FS:PAGE]

　　（四）以“三城联创”为抓手，推进区域性中心城市建设。

　　深入开展“三城联创”，大力推进基础设施建设，巩固和提升区域性中心城市地位。

　　高度重视规划编制工作。发挥规划的龙头作用，加快城市总体规划和土地利用总体规划修编，完善控规、专规、详规等相配套的规划体系，增强规划的科学性和指导性。

　　加快构建立体交通网络。加快武当山机场前期工作进程，为争取立项审批作准备。确保襄渝铁路二线投入运行，开工建设十宜铁路，加快十运、郑十铁路的前期工作。着力做好郧十和谷竹高速公路前期工作，开工建设十房、十白高速公路，新建白浪至武当山一级路，确保武神公路建成通车，完成兴界路、郧漫路等干线公路改造，加快推进郧县汉江二桥、丹江习均大桥建设。启动丹江口库区和武当山旅游码头建设。

　　大力提升城市功能。一是精心规划设计，把东风大道打造成迎宾大道，充分展示城市形象。二是续建上海路和凯旋大道二期，扩建车城西路，推进北京路南通道工程建设，打通重庆路和天津路的连接线，缓解中心城区交通压力。三是动工兴建建设二路，积极筹建发展大道、风神大道，加快西部万亩工业园区与市中心区连接线和外环通道建设。四是实施城区第三批背街小巷改造工程，分期升级改造人民路、公园路人行道，建设10座垃圾中转站，做好120万千瓦热电联供项目前期工作，开工建设天然气项目，提高人居环境质量。五是实施城中村改造工程，改善城市整体面貌。

　　切实加强城市管理。做好国家环保模范城市申报工作，加快国家卫生城市创建步伐。落实四级管理体制和门前三包责任制，广泛推行路长制，继续开展市容环境卫生等十个专项整治，标本兼治“脏乱差”现象。进一步加强绿化、美化、亮化工作，努力提升城市品位。

　　着力提高文明创建水平。以养成教育为突破口，提高市民文明素质。以《全国文明城市测评体系》为标尺，深入开展达标竞赛活动，以竞赛促创建。以文明新村为重点，推进农村文明创建活动，力争涌现一批文明村镇。强化全民国防教育，全面落实国防动员各项任务，加强人民防空和民兵预备役建设，广泛开展“双拥”活动，全面落实优抚安置政策，服务国防和军队建设。

　　加强生态建设与环境保护。继续加强退耕还林、长防林、天然林保护、重点生态公益林等工程建设，完成人工造林30万亩，进一步提高森林覆盖率。加快《丹江口库区及上游水污染防治和水土保持规划》项目实施，推进神定河和泗河污水处理厂、东部和西部垃圾场及各县市污水处理厂建设，加快泗河流域水生态整治修复试点建设步伐，确保库区水质保持在国家二级标准以上。加强农村污染治理和环境整治，进一步改善农村居住环境。启动“绿色公交”、“绿色校园”等工程，在城区推广清洁型汽车，推行乙醇汽油等新技术，根治公交尾气污染。加大金属冶炼、纺织等企业技术改造力度，继续淘汰落后产能，推广应用节能新技术、新产品。

　　（五）以体制机制创新为动力，着力推进改革开放。

　　继续深化各项改革。一是推进农村改革，积极稳妥推进农村土地管理制度改革，加快推进集体林权改革和农业“小三场”改革，探索土地、山场、林地、水面向种养能手、经营大户、龙头企业集中的有效途径，鼓励发展适合农村特点的各种微型金融服务。二是继续推进工业、商贸企业改革，加快企业改制步伐，力争毛巾厂、文贸公司等特困企业改制取得实质性进展。进一步完善企业国有资产监管体制，依法管理好国有资产，确保国有资产保值增值。三是深化城市建设投融资体制改革，加强城投公司融资平台建设。四是推进财税改革。继续深化财政四项改革，认真做好增值税转型、资源税等新一轮税收改革，积极应对税改给财政增收带来的困难，加强收入征管，严格控制一般性支出，确保公务购车用车、会议和接待费用、出国（境）经费等支出零增长。

　　提高招商引资实效。一是强化招商意识，健全激励机制。把招商引资作为抓项目保增长的总抓手，作为部门的主职责，千方百计引进资金和项目。建立直接有效的考核奖励机制，重奖招商有功人员，最大限度地调动干部群众招商积极性。二是调整主攻方向。将招商引资重点放在受金融危机影响小的国家和地区，放在北上西进的长三角、珠三角企业，放在国内同行业的领军企业，放在新材料、新能源、环保设备、农业产业化、旅游及服务外包等热门领域和前沿项目上。发改委、经委、商务局、农办等主要经济部门，每年要分别引进3-5个过亿元的工业项目、过5千万元的商贸项目、过千万元的农副产品加工项目。三是创新招商方式。重点采取产业链招商、园区招商、以商招商、代理招商和小分队招商等方式，提高引资整体水平。同时，强化招商项目督办服务，切实提高合同履约率和资金到位率。

　　广拓渠道扩大出口。充分利用广交会、东博会等商务会展平台，支持东风实业、双星东风、世纪中远等重点企业拓展拉美、非洲、中东等新兴国际市场，加强农副产品出口基地建设，优化出口商品结构，扩大出口规模。

　　（六）以改善民生为核心，推进和谐社会建设。

　　优先发展科教事业。继续重视科普工作，引导人民群众崇尚科学、破除迷信、消除陋习，改进生活方式，提高生活质量和水平。深入推进创新型城市建设，进一步增加科技投入，积极申报国家和省科技型中小企业创新基金，确保胚胎干细胞研究重点实验室通过省验收，加快高新区孵化器、湖北汽车传动与数字化控制重点实验室、模具工程技术中心等科技创新平台建设步伐，大力推进汽车及零部件技术创新示范区和科技创新示范县市、示范村、示范基地建设，确保新增高新技术企业20家。

　　继续落实“两免一补”政策，完善城乡贫困生救助体系，做好流动人口子女和农村留守学生教育工作，解决农村90%以上寄宿制学校“吃新鲜菜难、如厕难”和75%寄宿制学校的“洗澡难”问题，推进北京路初中建设，筹办武当国际武术学院，加快郧阳师专等高校迁扩建进程，确保学前教育普及率提高到70%，小学、初中辍学率控制在1%和3%以内，稳定中职学校招生规模，中职生就业率保持在96%以上。

　　繁荣文体事业。继续推进乡镇综合文化站、农家书屋、文化信息资源共享、农村数字化电影放映、农民体育健身等文体工程建设，广泛开展全民健身运动。完成第三次文物普查，抓好文物抢救和文化遗产保护工作。深化文化事业单位改革，推进公共文化服务体系建设，满足群众不同层次的文化需求。进一步推动有线电视数字化、数字电视移动化进程，提高广播电视覆盖率。推进卫生事业改革和发展。认真落实国家医药卫生体制改革方案，积极稳妥地推进卫生体制改革。加大农村卫生投入，进一步健全农村三级卫生服务体系。切实加强新型农村合作医疗的组织管理，确保农民参合率92%以上。探索建立市级医院带县（院）挂乡（院）医疗帮扶制度，实现医疗资源共享，努力缓解群众看病难、看病贵问题。做好人口与计生工作，确保人口出生率等指标控制在省定目标以内，建成国家、省计生优质服务先进县市区1-2个。

　　切实做好就业和社会保障工作。多渠道开发就业岗位，着力推进城乡统筹就业，鼓励自主创业、自谋职业和灵活就业，确保城镇新增就业3.5万人。扩大社会保险覆盖面，确保扩面新增6万人。进一步加强城乡救助体系建设，新增农村低保人口3万人，适当提高城乡居民最低生活保障标准。大力推进城乡保障性安居工程建设，力争15万平方米经济适用房和廉租房竣工，解决城镇低收入家庭住房困难问题；继续实施农村危房改造工程，逐步解决农村困难群众住危房问题；加大住房公积金贷款力度，增强中低收入家庭购房能力。加强农村福利院规范化管理，争取开工建设市儿童福利院和流浪未成年人保护中心。着力做好老龄工作，发展慈善事业。

　　全力维护社会稳定。积极组织生产和重要物资的调运，保证重要消费品不断档、不脱销。加强价格监管，依法打击囤积居奇、哄抬物价等违法行为，规范市场秩序，保障消费者的合法权益。加大医疗器械、药品、食品市场监管力度，保障人民群众使用药品和食品安全。强化安全生产监管，全面排查和治理重大安全隐患，坚决防止和遏制重特大事故发生。深入推进平安十堰建设，健全社会治安防控体系，依法打击违法犯罪活动，增强人民群众安全感。认真做好信访工作，畅通民情民意诉求渠道，加大矛盾纠纷排查调处力度，注重从源头疏导民怨、化解矛盾。

　　各位代表！努力为人民群众办实事、办好事，是各级政府应尽的职责。这里，我代表市政府郑重承诺，今年将继续办好十件实事，解决一批群众关心、社会关注的热点难点问题。一是加快推进城镇居民基本医疗保险工作，力争参保率达到80%。二是继续提高基层医疗机构服务水平，支持20个乡镇卫生院和200个村卫生室提升服务能力，支持城区15个社区卫生服务机构基础设施和服务功能建设。三是完善城乡社会服务体系，建设城市社区综合服务大厅33个、农村村级综合服务站100个。四是加快困难群众危房改造，基本解决市郊区农村五保户、低保户、优抚对象家住危房问题。五是继续抓好农村扶贫工作，完成扶贫搬迁和生态移民2750户1.1万人。六是切实抓好农村水利设施建设，完成2座中型水库、21座小型水库除险加固工程，解决农村安全饮水15万人以上。七是继续推进农村交通设施建设，新建通村水泥路1500公里，建设农村候车亭180个、招呼站300个。八是进一步改善城区生活环境，兴建山体游园和街头游园各3个；建设人行天桥3座；再建公厕10座。九是加强基层文化设施建设，新建村级农家书屋400家。十是创新“阳光工程”培训，完成农村非农技能培训和农业实用人才培训1.5万人。

　　（七）以提高执行力为着力点，全面加强政府自身建设。

　　推进民主政治建设。主动接受人大监督、政协监督和社会监督，认真做好议案、建议和提案的办理工作，切实提高办结满意率。完善重大决策专家论证、社会听证和合法性审查制度，促进行政决策民主化、科学化。推行政务公开，完善厂务公开、村务公开和公共事业单位办事公开制度，发挥市长热线、政府门户网站等媒介作用，保障群众的知情权、参与权、表达权和监督权。

　　坚持依法行政。扎实推进“五五”普法工作，深入贯彻《国务院关于加强市县政府依法行政的决定》，规范行政执法行为，坚决纠正多头执法、执法不公，严厉查处执法违法行为。

　　加强廉政建设。全面落实党风廉政建设责任制，严格执行领导干部廉洁从政各项规定。规范财政转移支付、政府采购、国有资产交易等公共资源管理权力运行，确保阳光行政。强化审计监督和行政监察，严肃查处一批群众反映强烈的企业改制、惠民补贴资金发放、工程建设、教育收费、医疗服务和医药购销、环境保护、行政权力运作等方面的腐败行为。狠刹赌博风、玩乐风、吃喝风，进一步转变干部作风。坚持艰苦奋斗、勤俭节约，切实降低行政成本。

　　着力建设效能政府。积极推进行政管理体制改革，健全政府职责体系，切实增强部门主体责任意识，增强工作主动性和创造性，增强协调配合能力，确保政令畅通。推进行政服务中心和部门服务窗口建设，进一步减少行政审批事项，规范审批程序，推行网上审批和办理服务，推进行政审批电子监察，切实提高审批效率。扎实开展政风行风评议，完善行政效能投诉受理机制，着力解决行政慢作为、不作为、乱作为问题。广泛推行“一线工作法”，积极服务企业渡难关、求发展，服务农民工实现再就业，服务招商引资创优环境。建立科学考评体系，完善工作量化考核办法，严格绩效考评，提高政府工作效能。

　　各位代表！新的一年，政府工作任务繁重、责任重大。让我们在市委的正确领导下，在市人大、政协的监督支持下，紧紧依靠全市人民的智慧和力量，以更加积极有为的精神状态、更加务实高效的工作作风、更加扎实有力的工作举措，开拓进取，迎难而上，为圆满完成全年各项目标任务，实现经济社会发展的新跨越而努力奋斗！
